# FACE-Q Patient Report-Assisted Subjective and Objective Evaluation of Blepharoplasty Outcomes Using Two Different Suturing Techniques: A Randomized and Patient-Blinded Pilot Study

**DOI:** 10.1007/s00266-023-03339-6

**Published:** 2023-05-01

**Authors:** Reinhard Told, Eva Placheta-Györi, Birgit Lackner, Andreas Kuchar, Jonas Brugger, Ursula Schmidt-Erfurth, Roman Dunavölgyi

**Affiliations:** 1 Department of Ophthalmology and Optometry, Medical University of Vienna, Vienna, Austria; 2 Department of Plastic and Reconstructive Surgery, Medical University of Vienna, Vienna, Austria; 3 Center for Medical Statistics, Informatics, and Intelligent Systems (CeMSIIS), Medical University of Vienna, Vienna, Austria

**Keywords:** Blepharoplasty, Suture, FACE-Q eye module

## Abstract

**Background:**

/Objectives

To compare two suturing techniques in patients undergoing upper eyelid blepharoplasty by using the FACE-Q™ Eye Module questionnaire to assess patient-reported outcomes and by blinded Likert-scale gradings of two experienced surgeons.

**Methods:**

90 patients undergoing bilateral blepharoplasty were randomly assigned to a suturing technique (running cutaneous or subcuticular closure) using Prolene 6.0. Patients completed the FACE-Q eye module questionnaire before surgery and 7 days and 3 months after surgery. Further, two trained oculoplastic surgeons assessed the outcome. FACE-Q ratings were RASCH-transformed, and linear models were fitted for appraisal and satisfaction results. Intraclass correlation coefficient (ICC) was calculated to assess the surgeons’ rating agreement.

**Results:**

There was no statistically significantly difference in patients’ FACE-Q self-assessments regarding satisfaction with eyes and appraisal of upper eyelids between the two suturing techniques investigated, both 7 days and 3 months after blepharoplasty. The more content the patient at baseline, the less the increase in satisfaction after 3 months. There was good agreement between blinded graders in outcome assessment expressed by an ICC of 0.86. Dry-eye symptoms increased after surgery, independent of the suturing technique, patient age or sex.

**Conclusion:**

In conclusion, this study shows that post operative patient satisfaction is independent of suturing technique, but depends on baseline FACE-Q reports. These findings are valuable in patient communication and selection and are in line with observer-based assessments.

**Level of Evidence III:**

This journal requires that authors assign a level of evidence to each article. For a full description of these Evidence-Based Medicine ratings, please refer to the Table of Contents or the online Instructions to Authors http://www.springer.com/00266.

**Supplementary Information:**

The online version contains supplementary material available at 10.1007/s00266-023-03339-6.

## Background

Upper blepharoplasty, where excess skin of the upper eyelid is resected, is the most common procedure in facial plastic surgery and periorbital surgery. Data of the International Society of Aesthetic Plastic Surgery (ISAPS) show that in 2021, blepharoplasty was the fifth most common plastic surgery procedure in the United States, with 150,000 operations performed. [1]

Surgical outcomes and treatment success are mainly assessed using objective measurements such as postoperative photographs and perimetric visual field measurements, [2] as well as observer-based ratings such as the Vancouver Scar Scale.[3, 4]

However, patient-reported outcome measures determining patient satisfaction and health-related quality of life are essential for comprehensive assessments.[5, 6] The need for a systematic approach was recently fostered by the inability of a large-scale study to perform a meta-analysis assessing over 4000 studies.[7] This clearly highlights the heterogeneity in this field of research. This can be traced back to the multitude of classification schemes used, which have emerged mostly from burn scars and focus mainly on scar formation.[8]

There is well-documented evidence that validated patient-reported outcome measures are superior to *ad hoc* questionnaires.[9] Studies have shown that disease-specific instruments measuring health-related quality of life are essential because conditions affecting the face are not sufficiently captured by general quality of life assessments.[9, 10]

Recently, Klassen and colleagues developed the FACE-Q scales [11, 12], which measure patient satisfaction after aesthetic surgical procedures, and a subset specifically aimed at patients undergoing periorbital procedures, the FACE-Q Eye Module[13]. The FACE-Q Eye Module has 4 scales that measure appearance of the eyes, upper and lower eyelids, and eyelashes. The module also includes a checklist that assesses complications after blepharoplasty.[13] To date, the FACE-Q Eye Module is the most comprehensive, validated, and established patient-reported outcome instrument available to evaluate patients undergoing blepharoplasties.

In this study, we aimed to investigate the differences between two common techniques of skin closure: running cutaneous and subcuticular sutures. The effects on postoperative patients’ satisfaction, appraisal, and adverse effects were investigated using the FACE-Q Eye Module [13]. Patient-based gradings were subsequently compared with observer-based outcome assessments.

## Methods

The Ethics Committee of the Medical University of Vienna, Vienna, Austria approved the protocol of the present study (ClinicalTrials.gov: NCT04924972), which was conducted in adherence to the Declaration of Helsinki including current revisions and the Good Clinical Practice (GCP) guidelines. Written informed consent was obtained from all patients participating in this study before study inclusion.

In this randomized, patient- and observer-blinded cohort study, we included 90 female or male patients, 18 years or older, with bilateral dermatochalasis.

Patients were not included if any diseases affecting wound closure or healing were present. Further, untreated hypertension or metabolic syndrome, smoking, asymmetric brow ptosis, and any other disorder of the eyelid or sequelae after facial paresis were considered an exclusion criterion. A known allergy or adverse reaction to any substance used was also considered an exclusion criterion.

### Study Visits and Examinations

Patients included in this study were scheduled for three visits. Visit 1 was the day of surgery (baseline), visit 2 the day of suture removal 7 days after surgery, and visit 3 the day 3 months (± 2 weeks) after surgery.

#### FACE-Q Questionnaire

The FACE-Q questionnaire [14] is a collection of 20 questions that focus on cosmetic eyelid surgery and evaluates the appraisal of upper eyelids, satisfaction with eyes and adverse effects after eyelid surgery. Each FACE-Q scale presents multiple items that can be scored on a 4-point Likert scale, after which a sum score is calculated. The scores of each scale are RASCH-transformed to a range from 0 to 100. Patients completed a FACE-Q questionnaire at all three visits. A mirror was provided to patients if needed.

#### Photographs

Standardized photographs were taken at all visits. Facial photographs in frontal, lateral, and oblique views, with a downward, straight, and upward gaze, were recorded.

#### Overall Cosmetic Evaluation

Independent surgeons graded the overall cosmetic appearance based on the standardized photographs on a Likert scale from 1 to 4; 1 represents excellent wound healing (scar matches surrounding skin), whereas 4 represents poor healing and scar formation (scar does not match surrounding skin). The mean values were determined for each study visit and used for subsequent statistical calculations.

#### Randomization

Block randomization (Sealedenvelope.com) was performed to assign patients to running cutaneous or subcuticular sutures, the two standard procedures for wound closure after upper eyelid blepharoplasty.

#### Standard Surgical Procedure

A standard surgical procedure was performed for excessive upper eyelid skin removal. A standardized aseptic procedure for skin disinfection (Octenisept), local anesthesia (Xylanaest 2% + epinephrine 1:200,000), pinch technique for assessing skin to be excised, scalpel, bipolar cautery, and skin closure as previously randomized with 6-0 Prolene sutures (Ethicon, Prolene™, Polypropylen 6-0, Needle: C-1 13mm 3/8c) were performed. Steri strips were used until suture removal for patient blinding. Vitamin A ointment two times daily was prescribed up to the month 3 visit.

#### Main Outcome Variable

FACE-Q patient report outcomes after blepharoplasty using different suturing techniques.

## Statistical Analysis

Patient demographics were reported descriptively. FACE-Q ratings (0–4) were transformed to the equivalent RASCH score ranging from 0 to 100. Linear models were computed to investigate whether the change in RASCH score after surgery depended on the suturing technique. The difference in RASCH score at month 3 after blepharoplasty and baseline was defined as the dependent variable and the study group as well as the baseline RASCH score as the explanatory variables. The baseline RASCH score was centered to obtain reasonable estimates of a baseline difference for an average baseline score. Linear models were fitted separately for the RASCH satisfaction score and RASCH appraisal score.

Estimates, 95%-confidence intervals and *p*-values were computed for the average difference within the entire study cohort, as well as the change for each unit increase in the baseline score and contrast estimates between the two study groups. As a sensitivity analysis, scores of patients assessed by both surgeons were averaged and the same models were calculated using the average value. Intraclass correlation coefficient (ICC) was calculated to assess the surgeons’ rating agreement. Statistical analyses were performed using R, version 3.6.1 or higher.

## Results

Of the 90 patients included, 88 successfully completed this study. Two patients were lost to follow-up after suture removal. Consequently, the results are based on 88 patients. Patients were aged between 30 and 86 years (mean 62 ± 12 years). 80% of our patients were women (70), 20% were men (18). See Table 1 for distribution within groups.

**Table 1:** Baseline characteristics within groups

v	Running continuous suture	Subcuticular suture	*p* value
*n*	45	43	
Age (mean years ± SD)	59 ± 12	64 ± 11	0.06
female/male (*n*)	36/9	34/9	0.91

*SD* standard deviation; *n* number

### Patient FACE-Q Assessment

#### Satisfaction with eyes

**Fig. 1:**
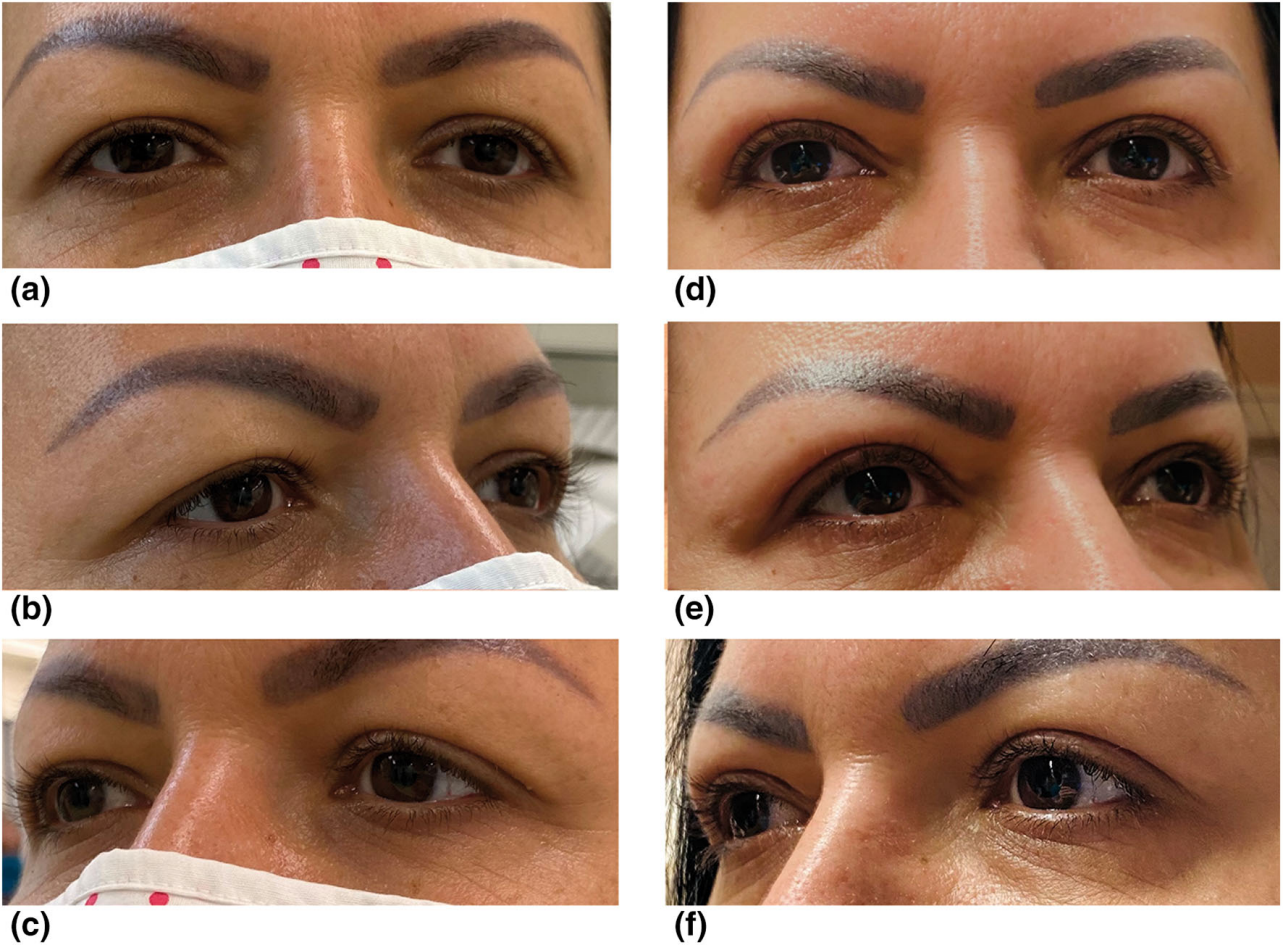
Patient reporting the highest change in RASCH score based on the FACE-Q satisfaction assessment. This was a 45-year-old woman with disturbing dermatochalasis on both eyes (Fig. 1a–c). She underwent dermatochalasis operation and was reassessed 3 months thereafter (Fig. 1d–f). Wound closure was performed in a continuous fashion

**Fig. 2:**
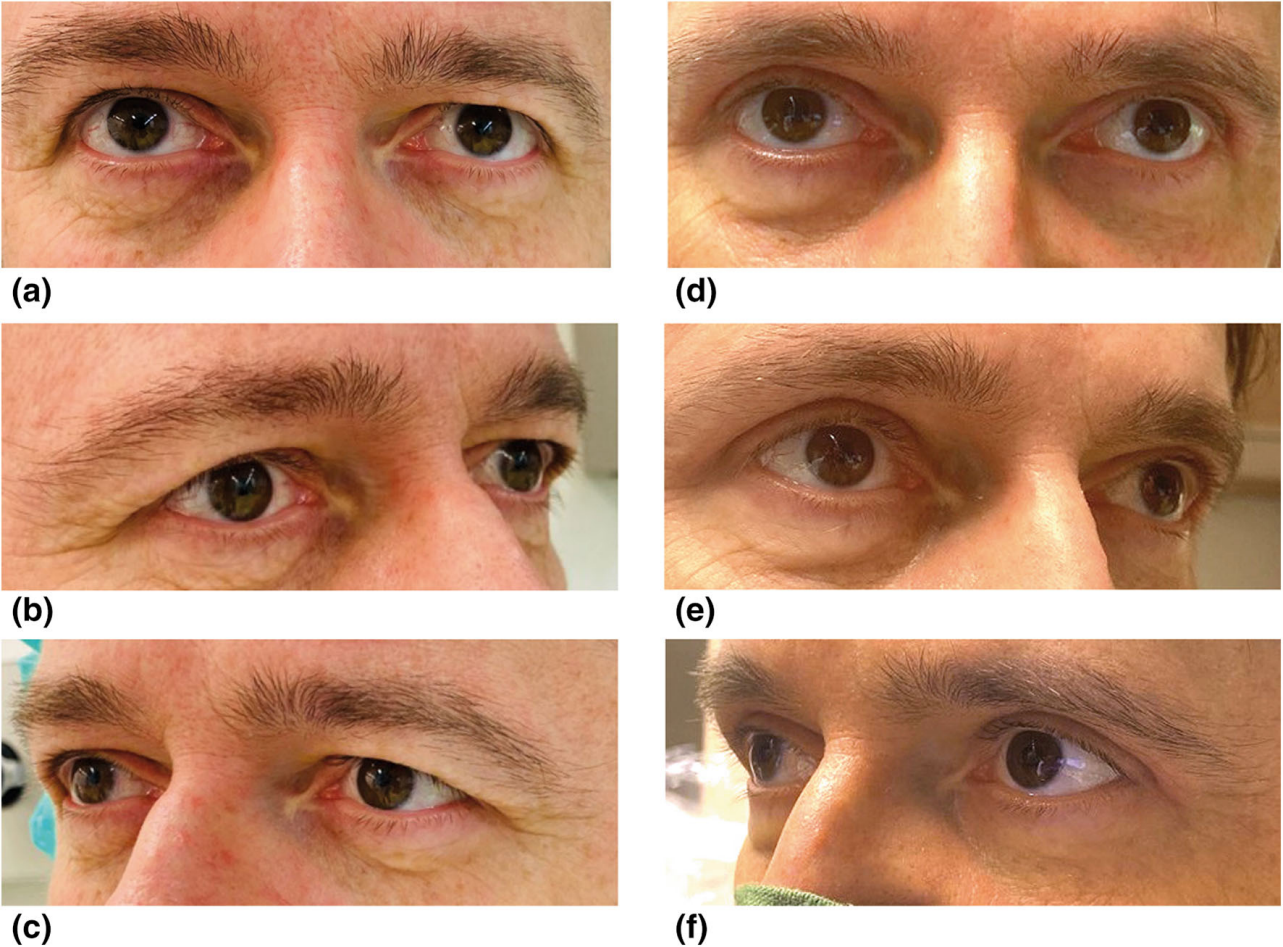
Patient reporting the least change in RASCH score based on the FACE-Q satisfaction assessment. This was a 50-year-old man with disturbing dermatochalasis on both eyes (Fig. 2a–c). He underwent dermatochalasis operation and was reassessed 3 months thereafter (Fig. 2d–f). Wound closure was performed in a continuous fashion

The results suggest that patients’ satisfaction with eyes on average statistically significantly increases by 50 RASCH points (*p* < 0.001) regardless of the type of suturing technique used. Consequently, no significant differences between suturing techniques (*p* = 0.8) were observed regarding satisfaction with eyes (see Table 2). The difference between satisfaction at month 3 and baseline satisfaction was statistically calculated to be 0.80 (*p* < 0.001) lower for each RASCH point increase in baseline score. In other words, the more content the patient was at baseline, the less the increase in satisfaction after 3 months. Figure 1 shows the patient whose satisfaction score increased the most, 100 points—which is the maximum possible. Figure 2 shows the patient whose satisfaction score increased the least (4 points).

#### Appraisal of upper eyelids

**Table 2:** Patient FACE-Q assessment: Comparison between groups regarding FACE-Q RASCH score based on ‘Satisfaction With Eyes’ and ‘Appraisal Of Upper Eyelids’ questionnaire at baseline (BL) and after 3 months (M3)

	Group	RASCH Score (0–100 a.u.)		*p*-value
		Running continuous suture	Subcuticular suture	
Satisfaction with eyes	BL	34 ± 19	32 ± 20	0.80
	M3	83 ± 23	84 ± 17	
	*p value*	< 0.001		
Appraisal of upper eyelids	BL	26 ± 17	27 ± 23	0.44
	M3	92 ± 15	89 ± 15	
	*p value*	< 0.001		

Patients’ appraisal of upper eyelids increased by 65.12 RASCH points (*p* < 0.001) on average regardless of the type of suturing technique used. No significant differences between suturing techniques (*p* = 0.44) were observed (see Table 2). The difference between appraisal at month 3 and baseline appraisal was statistically calculated to be 0.98 (*p* < 0.001) lower for each RASCH point increase in baseline score. This indicated that the higher the baseline RASCH score on how patients appraised their upper eyelids, the less the increase in the score seen 3 months later.

### Adverse Effects: Eyes

FACE-Q adverse effects reported by patients at day 7 included scar formation, eye irritation, excessive tearing, hollowing of eyes, and difficulty closing eyes. In addition, two surgeons rated infection, milia, and wound dehiscence 7 days after blepharoplasty. Overall, the before-mentioned adverse effects occurred in 37% (42) of all patients. There was no statistically significant difference between wound closures, either 7 days or 3 months after upper eyelid blepharoplasty regarding adverse effects. See Table 3 for details; see supplemental Fig. 1 for ‘scar’-pictures. Surgeons did not rate adverse effects to be severe. However, two patients in the continuous closure group and one in the subcuticular running group had mild milia 7 days after blepharoplasty. One patient in the subcuticular running group and one in the continuous closure group had a mild infection. None of the patients needed additional treatment after suture removal. There was no wound dehiscence in either group. ICC for surgeons’ ratings was 0.72. In this study, about 50% of patients had dry eyes at day 7, as reported on the FACE-Q questionnaire. This number decreased to 40% after 3 months. There was no statistically significant difference between the groups, either at day 7 or month 3 after blepharoplasty (see Table 3).

**Table 3:** Patient-reported FACE-Q as well as additional adverse effects recorded by two surgeons based on photographs, which were scored on a 4-point Likert scale (not at all, a little, moderately, extremely)

FACE –Q adverse effects	*p*-value		Delta	
	D7	M3	M3-D7	
			Continuous *n*=45	Subcuticular *n*=43
How your eyelid scars look (obvious, noticeable, uneven)?	0.92	0.85	− 0.09	− 0.16
Dry eyes?	0.53	0.27	− 0.04	− 0.16
Eye irritation (e.g., redness, itching)?	0.92	0.52	− 0.60	− 0.53
Excessive tearing?	0.38	0.9	− 0.31	− 0.53
Your eyes looking hollowed out?	0.63	0.24	− 0.04	0.00
Difficulty closing your eyes?	0.52	0.89	− 0.18	− 0.07
Additional Adverse Effects				
Infection?	0.94			
Milia?	0.62			
Wound dehiscence?	n/a			

The *p*-value represents a comparison between groups based on suturing technique: running cutaneous and subcuticular closing. Delta represents the difference between reports 3 months and 7 days after blepharoplasty. Negative delta values represent a decrease in severity over time of the items recorded. n/a = not applicable

### Surgeons’ Overall Cosmetic Evaluation

The surgeons’ overall cosmetic evaluation shows a mean 1-point decrease from 2 at day 7 to 1 at 3 months. This was the case in both groups, continuous and subcuticular closing, and represents excellent wound healing 3 months after blepharoplasty. The change was not statistically significantly different between the groups (*p* = 0.83). The overall cosmetic evaluation (1–4 Likert scale) shows good agreement between surgeons (ICC 0.86). A model assessing the influence of age and sex based on the median of the surgeons’ ratings reveals that at day 7, age has a statistically significant influence on the surgeons’ overall cosmetic evaluations (*p* = 0.04), whereas sex has no statistical influence (*p* = 0.45). However, this changes at month 3, where sex (*p* = 0.03), but not age (*p* = 0.54), shows a statistically significant influence on the surgeons’ evaluations.

## Discussion

As blepharoplasty is the most commonly performed facial plastic and periorbital procedure [1], numerous studies have tried to quantify surgical outcomes. Fearmonti et al. published a review on scar scales and scar measuring devices and clearly outlined that these scales are mainly based on burn scars or evaluations after breast cancer surgery.[8]

Other studies applied eyelid-specific scar scales. For example, a non-validated *ad hoc* questionnaire shows that all patients were satisfied with the postoperative results and would choose to undergo the procedure again. Only seven very broad domains were graded by the patients on a 5-point Likert scale.[15] The Blepharoplasty Outcome Evaluation (BOE), consisting of six items analyzing functional, aesthetic, and social aspects of the eyes (5-point scale), the Derriford Appearance Scale (DAS59), which measures psychological distress and the effects on daily life associated with self-consciousness of appearance, four visual analog scales, and the Glasgow Benefit Inventory, which is a non-specific instrument that assesses patient satisfaction after a procedure were used.[16] The multitude of instruments applied and associated time invested by patients renders this protocol unsuitable for clinical practice.

In recent years, validated questionnaires have been developed specifically for patients undergoing facial procedures. With the development of the FACE-Q scales, patient-reported outcome measures for surgical and nonsurgical aesthetic facial procedures have become available. These, like widely used BREAST-Q and BODY-Q scales, were developed according to the international guidelines for patient-reported outcome instrument development.[12] The FACE-Q [11, 12] questionnaire analyzes patient satisfaction and quality of life in five subscales: Psychological Wellbeing, Social Function, Satisfaction with Decision to Have Treatment, Satisfaction with Outcome of Treatment, and Early Life Impact of Treatment.[12] The FACE-Q Eye Module has four scales that measure the appearance of the eyes, upper and lower eyelids, and eyelashes. In this study, we focused on the appearance of and satisfaction with the upper eyelids. Additionally, two independent surgeons rated wound infections, milia, as well as wound dehiscence 7 days after blepharoplasty based on photographs [4].

Patient 'Satisfaction with Eyes' assessment focuses on the shape as well as how attractive, alert, open, bright-eyed, nice, and youthful patients’ eyes appear.[13] Our results suggest that the worse the initial patient satisfaction, as measured in FACE-Q RASCH score, the higher the patient satisfaction 3 months after upper eyelid blepharoplasty. This represents an indirect relation of baseline patient satisfaction to postoperative satisfaction scores. On average, satisfaction statistically significantly increased by 50 RASCH points (0–100 scale). This was independent of the suturing technique used (running continuous, subcuticular suture).

The strong increase in satisfaction mirrors surgeons’ overall cosmetic evaluation, showing minimal scar formation 3 months after blepharoplasty. This rating was independent of the suturing technique used. However, age and sex had an influence on the surgeons’ perception, as age had a statistically significant influence at day 7 ratings, whereas sex had a statistically significant influence at 3 months after blepharoplasty. The postoperative increase in patient satisfaction is further in accordance with a previous study. Domela-Nieuwenhuis et al. found an increase in 48 FACE-Q RASCH points in a large multicenter trial including over 2000 patients.[17] However, the exact surgical procedure and suturing technique were not predefined. A systematic review of 26 studies has shown that surgical techniques do not influence patient satisfaction after blepharoplasty.[7] It has to be mentioned that these studies were mostly retrospective in nature, and satisfaction was assessed based on an analog scale or Blepharoplasty Outcome Evaluation (BOE).[16] Currently, data indicates that neither the suturing technique, as assessed in our study, nor suturing material [18] have a statistically significant effect on postoperative patient satisfaction. When drawing conclusions from these previous studies, it has to be kept in mind that not all the studies cited are based on the validated FACE-Q questionnaire.

FACE-Q ‘Appraisal of Upper Eyelids’ focuses on how various aspects of the upper eyelids may bother the patient. Consequently, a low initial RASCH score represents patients severely bothered by eyelid skin on lashes, saggy, droopy, or heavy eyelids, folds, tired or old looks due to the upper eyelids. Our results indicate that the lower the initial RASCH score, the stronger the increase in RASCH score 3 months after surgery. On average, the appraisal RASCH score increased by 65 points. This finding is independent of the suturing technique used. A large-scale study including over 2000 patients with a 50% response rate showed an average change of 47 RASCH score points.[17] The average score at inclusion was 44 and increased further to 91 after 6 months. This is different from our study, as our patient collective reached a pre-operative RASCH score of around 26 (see Table 2). The willingness of patients in different countries to undergo plastic surgery might explain this difference, as most of our patients undergo blepharoplasty as soon as visual field restrictions are disturbing.

Studies show that adverse effects occur rarely after upper eyelid blepharoplasties.[19, 20] Joshi et al. found that different types of suturing material induced considerable differences regarding milia, erythema, and scaring. In short, they found polypropylene sutures showed the lowest complication rates.[2] This bias can be ruled out in our study as only 6-0 Prolene sutures were used.

FACE-Q adverse effects reported by patients included scar formation, eye irritation, excessive tearing, hollowing of eyes, and difficulty closing eyes. In addition, two surgeons rated infection, milia, and wound dehiscence 7 days after blepharoplasty. Overall, the before-mentioned adverse effects occurred in 37% (42) of all patients. There was no statistically significant difference between wound closures, either 7 days or 3 months after upper eyelid blepharoplasty regarding adverse effects. See Table 3 for details; see supplemental Fig. 1 for ‘scar’-images taken three months after blepharoplasty. Surgeons did not rate adverse effects to be severe. However, three patients in the continuous closure group and two in the subcuticular running group were rated to have either mild milia or infection 7 days after blepharoplasty. None of the patients needed additional treatment after suture removal. There was no wound dehiscence in either group. Consequently, our analysis showed no statistically significant difference between groups regarding adverse effects.

A recent study comparing two surgical techniques (skin only and additional orbicularis oculi muscle excision) showed no statistically significant differences in FACE-Q adverse effects. Consequently, the surgical technique may potentially be ruled out as a source of adverse effects.[7] The exact time point of assessment has to be kept in mind when comparing studies for adverse effects.

A limitation of this study is that the patients included mostly presented with visual field restriction before undergoing blepharoplasty. Care is needed when extrapolating these results to patients undergoing upper eyelid blepharoplasty for cosmetic reasons only. Also, the findings only apply to the suturing material used. Consequently, there is still a need to gain insight as to whether our results are true for other suturing materials available. The strength of this study clearly is its assessment with validated patient-reported outcome measures, prospective design, and low dropout rate.

In summary, our study found no statistically significant difference between running cutaneous and subcuticular closing techniques regarding patient satisfaction and appraisal of upper eyelids 3 months after blepharoplasty. This study further demonstrated that satisfaction of pre-operatively unhappy/dissatisfied patients was more likely to increase compared with patients who reported themselves rather satisfied before blepharoplasty. Ratings by two graders showed good overall agreement.

## Conclusion

In conclusion, this study shows that post operative patient satisfaction is independent of suturing technique, but depends on baseline FACE-Q reports. These findings are valuable in patient communication and selection.

## Supplementary Information

Below is the link to the electronic supplementary material.

This image shows patients displayed in Fig. 2 (A–C) and Fig. 1 (D–F) with a downward gaze allowing the display of potential scars three months after blepharoplasty (TIFF 1142 KB)
