# Supplementary figures and images for: FACE-Q Patient Report-Assisted Subjective and Objective Evaluation of Blepharoplasty Outcomes Using Two Different Suturing Techniques: A Randomized and Patient-Blinded Pilot Study
Source: Aesthetic Plast Surg. 2023 May 1;47(4):1410–7. doi: 10.1007/s00266-023-03339-6 (PMC10390350; doi:10.1007/s00266-023-03339-6)

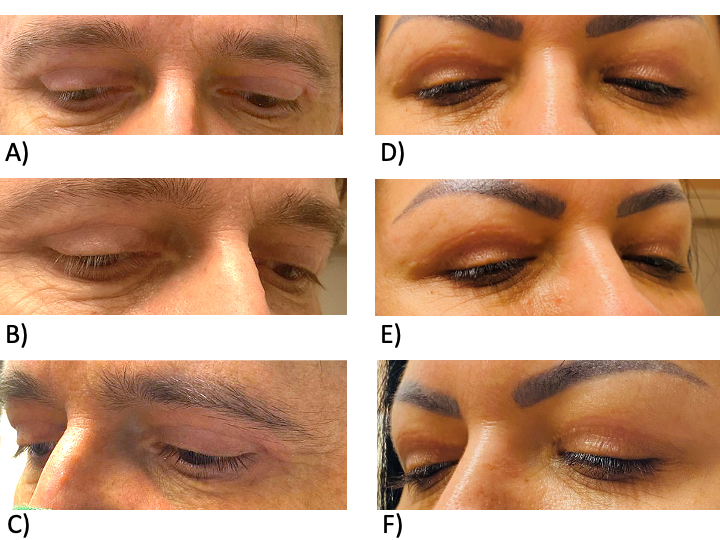

Supplement: Supplementary file 1 — This image shows patients displayed in Fig. 2 (A–C) and Fig. 1 (D–F) with a downward gaze allowing the display of potential scars three months after blepharoplasty (TIFF 1142 KB) [file 266_2023_3339_MOESM1_ESM.tiff]
